# Supplementary material for: Perceptual constancy of pareidolias across paper and digital testing formats in neurodegenerative diseases
Source: Heliyon. 2024 Nov 8;10(22):e40254. doi: 10.1016/j.heliyon.2024.e40254 (PMC11585718; doi:10.1016/j.heliyon.2024.e40254)
Supplement: Multimedia component 2 [file mmc2.docx]

**Supporting information**

**Suppl Figure-1. Correlation between pareidolia and MMSE scores for AD and DLB**


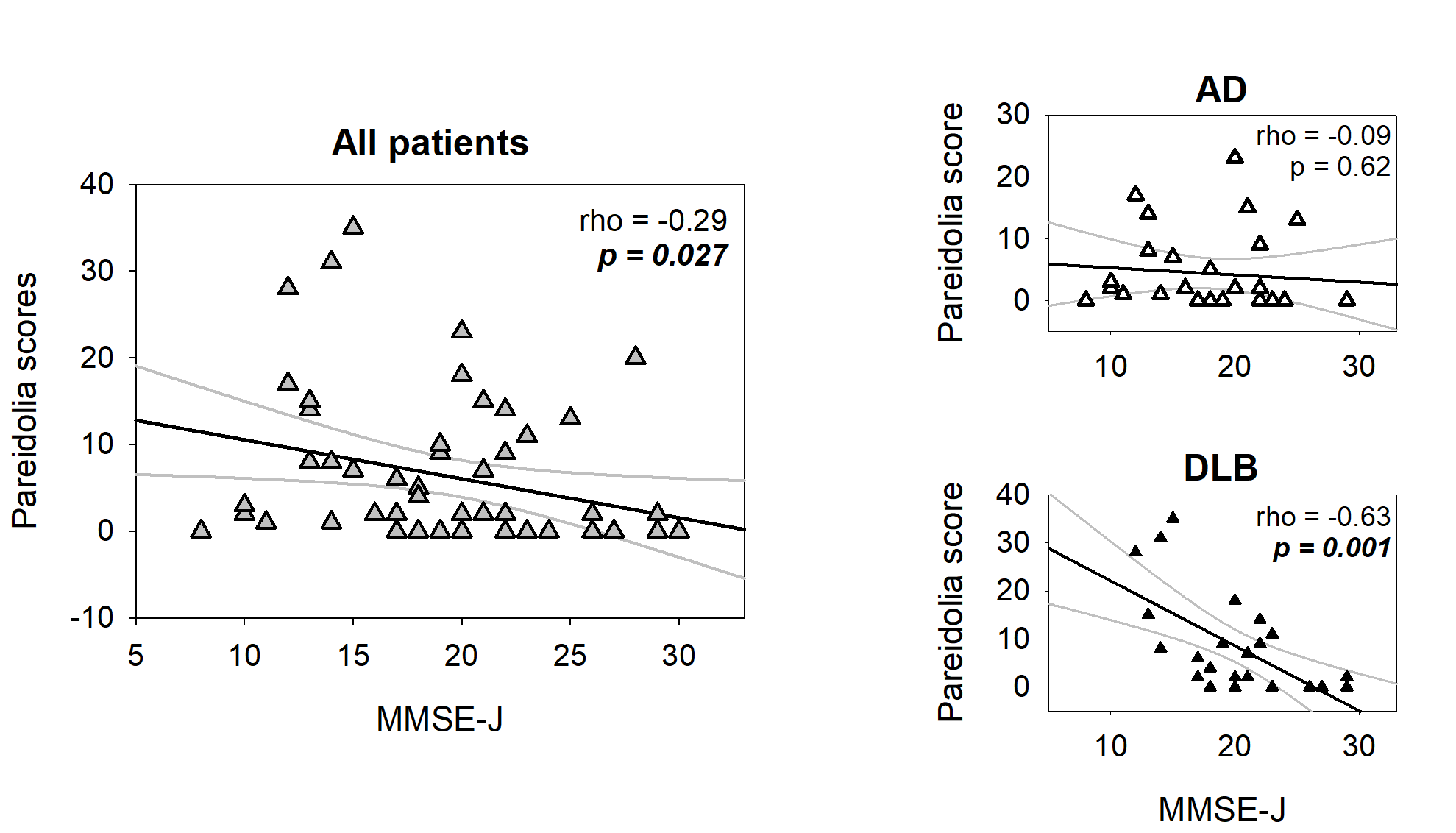


Suppl Figure-1. shows data with regression line in solid black with CI’s in solid gray, with Spearman’s correlation coefficient values within the graph. MMSE-J = Mini-mental state examination, Japanese version. DLB patients had a strong negative correlation between Pareidolia and MMSE scores contributing significantly to the patient group.
